# Inappropriately prescribed and over-the-counter antimicrobials in the Brazilian Amazon Basin: We need to promote more rational use even in remote places

**DOI:** 10.1371/journal.pone.0201579

**Published:** 2018-08-03

**Authors:** Abel Santiago Muri-Gama, Albert Figueras, Silvia Regina Secoli

**Affiliations:** 1 Universidade Federal do Amazonas −UFAM, Instituto de Saúde e Biotecnologia− ISB, Coari, Brazil; 2 Departament de Farmacologia, de Toxicologia i de Terapèutica, Universitat Autònoma de Barcelona, Fundació Institut Català de Farmacologia, Barcelona, Spain; 3 Escola de Enfermagem da Universidade de São Paulo, São Paulo, Brazil; University of Sydney, AUSTRALIA

## Abstract

**Background:**

Being aware of consumption patterns of antimicrobials is the first step in designing and implementing strategies to change behaviors and, thus, to reduce the occurrence of antimicrobial resistance. The present survey was carried out to identify and describe the use of antimicrobials without prescription in riverside dwellers of the Brazilian Amazon Basin.

**Methods:**

A cross-sectional study was carried out from a conglomerate stratified sample in the rural municipality of Coari, Amazonas State, Brazil, between April and July 2016. The survey was conducted in the riverside dwellers’ homes, and information was collected on all antimicrobials taken with and without medical or dental prescription for a 30-day period, together with indications of their use before the interview.

**Results:**

A total of 492 riverside dwellers were included in the present survey; 346 (70.3%) had taken at least one medication during the previous month, and 74 (21.3% of those taking a medicine) used an antimicrobial. Two-thirds of the patients treated with an antimicrobial (49; 66.2%) obtained it without a prescription. Additionally, one-third of the antimicrobials consumed by the study sample (25) were used for non-infectious or non-bacterial conditions.

**Discussion:**

The present survey showed not only that inappropriate use of antimicrobials is present in remote places such as the Amazon Basin, but also that one-third of those antimicrobials were taken to treat non-infectious or non-bacterial conditions. In addition to an unnecessary risk of adverse effects to the exposed populations, the inappropriate use of antibiotics without prescription helps to increase antibiotic-resistant strains. In the present case, this was happening near one of Latin America’s most important water supplies, which could contribute to the global impact of antimicrobial resistance.

## Introduction

Antimicrobial resistance (AMR) is one of the main challenges in the management of infectious diseases. The risks and potential health, social and economic consequences of AMR for humankind prompted an unusual action by the United Nations General Assembly, which recently committed to addressing this problem [1]. Many causes explain the development of resistance by microorganisms, a phenomenon that can appear naturally, but has become a global threat due to several factors, namely: (1) overprescription and misuse of antibiotics, (2) aggressive promotion by pharmaceutical companies, (3) underinvestment in infection control, (4) non-compliance by patients, and (5) weak hospital management practices [2]. In addition to the human use or misuse of antimicrobials (AMs), there are other important factors involved in the growth of AMR, such as the use of AMs in veterinary medicine and the presence of AMs in the environment. To address this, a One-Health initiative is being developed to break down barriers between the health of animals, humans, and the environment in order to better serve the cause [3].

Regarding the medical utilization of AM, misuse is a global problem that affects hospitals, outpatients, and long-term care settings [4], and involves all the actors and processes of the so-called therapeutic supply chain, from the market and overrepresentation of products containing AMs [5] to the prescription and use of these AMs, including self-medication and selling without prescription [6]. In Brazil, as in other Latin American countries, the dispensation of antimicrobials without prescription is frequent, despite a variety of national regulations forbidding it [7]. At present, up to 128 active ingredients should be sold only under prescription in all pharmacies in Brazil [8], following the WHO recommendations for fighting antimicrobial resistance as a public health threat [9]. In Brazil, inappropriate AM prescription, uses of counterfeit antibiotics, and self-medication have been described. Regarding self-medication, the main outcomes are usually underdosage, lack of adherence, and, in some cases, consumption of expired products; additionally, the lack of a reference microbiological laboratory in most Brazilian cities aggravates this situation [10].

The Amazon Basin is one of the most remote inhabited places on Earth. Along the Amazon River, an area of 7,500,000km^2^ including portions of eight Latin American countries is bordered by sparsely populated rainforest. The so-called “*ribeirinhos*” (riverside dwellers) descended from a mixture of indigenous people and immigrants from other Brazilian regions and live on the banks of the Amazon’s rivers and lakes [11]. In general, those inhabitants have low economic and educational levels and are usually fishermen and farmers cultivating small family plots. They live in isolated communities with limited access to urban areas: the only way to reach the city is sailing an average of 60 km–a four hour journey. Additionally, the health infrastructure is precarious, as the communities lack basic sanitation and health services.

A population study was carried out with the aim of analyzing the use of AMs with and without prescription among this population, as well as indications of their use.

## Methods

### Selection of survey populations

A population-based cross-sectional study was carried out among *ribeirinhos* living in the rural area of the Coari municipality, Amazonas State, Brazil, between April 1 and July 31, 2016. Coari is located in the Amazon central region and can be reached only by boat or small airplane. In 2015, the estimated population was 83,078 inhabitants; one-third (riverside dwellers) lived in rural zones disseminated along the Solimões River banks, covering an area of 59,976 km^2^with a very low population density (1.3 inhabitant per km^2^) [12, 13].

The conglomerate probabilistic sample without repositioning included in the present study represented 10,333 *ribeirinhos* aged more than 18 years-old distributed along the 135 riverside communities registered by the Secretaria Municipal de Saúde (SEMSA, Municipal Health Office). The sample was taken from population data based on the inhabitants registered in the Sistema de Informação da Atenção Básica (Basic Care Information System), a database that is updated monthly, so this is the most accurate population information in this remote region, and it was used for sampling.

The sample size was estimated after taking into account a self-medication prevalence of 50% with a 5% margin of error, and 95% confidence level, according to a previous general health survey in these communities [13]. An adjustment for finite population was made, and a 20% loss or non-completition was accepted. Therefore, the sample size was calculated (for an infinite population) as:

n=Zα22 (p·qE2)

- n = sample size

- Z_α/2_ = critical value for the desired confidence degree; in the present study, 1.96 (95%)

- E = margin of error; in the present study, ± 5% of the proportion of cases (absolute precision)

- p = proportion of the favorable outcomes in the population; a 50% self-medication proportion was used because it was the only proportion identified in a previous local study [14].

The sampling process was carried out in two steps: (1) a random selection of each riverside-dweller community in each region with a proportional probability according to each community population, and (2) random selection of the addresses in each sorted community.

The random selection of the residences was done using a ballot system: when we arrived at the community, the first house was selected, the second excluded, the third selected, and so on. In the sorted homes, all adults resident were interviewed. If the researcher reached the end of the community without filling the expected minimum number of homes for that community, the non-selected homes were included in order to achieve this number.

Thus, the estimated sample was 470 riverside-dwellers (out of a total population of 10,333 river dwellers aged ≥ 18) living in 24 communities distributed in eight areas between lakes and rivers in the rural zone of Coari. The regions were: Alto Solimões, Médio Solimões, Baixo Solimões, Coari Lake, Mamiá Lake, Copeá River, Piorini River and Codajás-Mirim River. The regional delimitation methodology used in the present study was the same as that used by the local healthcare system [13].

Although a 20% loss was initially anticipated, it did not in fact actually occur because the repositioning strategy was used. So, although the originally estimated sample included 470 inhabitants (374 plus 20% losses), after visiting all the communities, 492 persons had been actually interviewed. This final figure was maintained, as it increases the power of the study.

### Antimicrobial use survey

The information regarding health status and use of medicines was obtained by means of individualized interviews in the selected homes. In addition to the interview, the researchers asked to see the storaging places, package inserts and remaining drugs in each visited home (see S1 and S2 Files). The researchers assessed the use of AMs not prescribed by any physician or dentist for 30 days prior to the date of the interview.

### Ethical considerations

The present study was approved by the Research Ethics Committee of the Nursing School of the Universidade de São Paulo (Brazil) and the reference Nr. 33560914.0.0000.5392 was assigned. An informed consent was signed by all the included inhabitants. People appearing in the photos gave their consent to be portrayed in these images.

## Results

**Fig 1:**
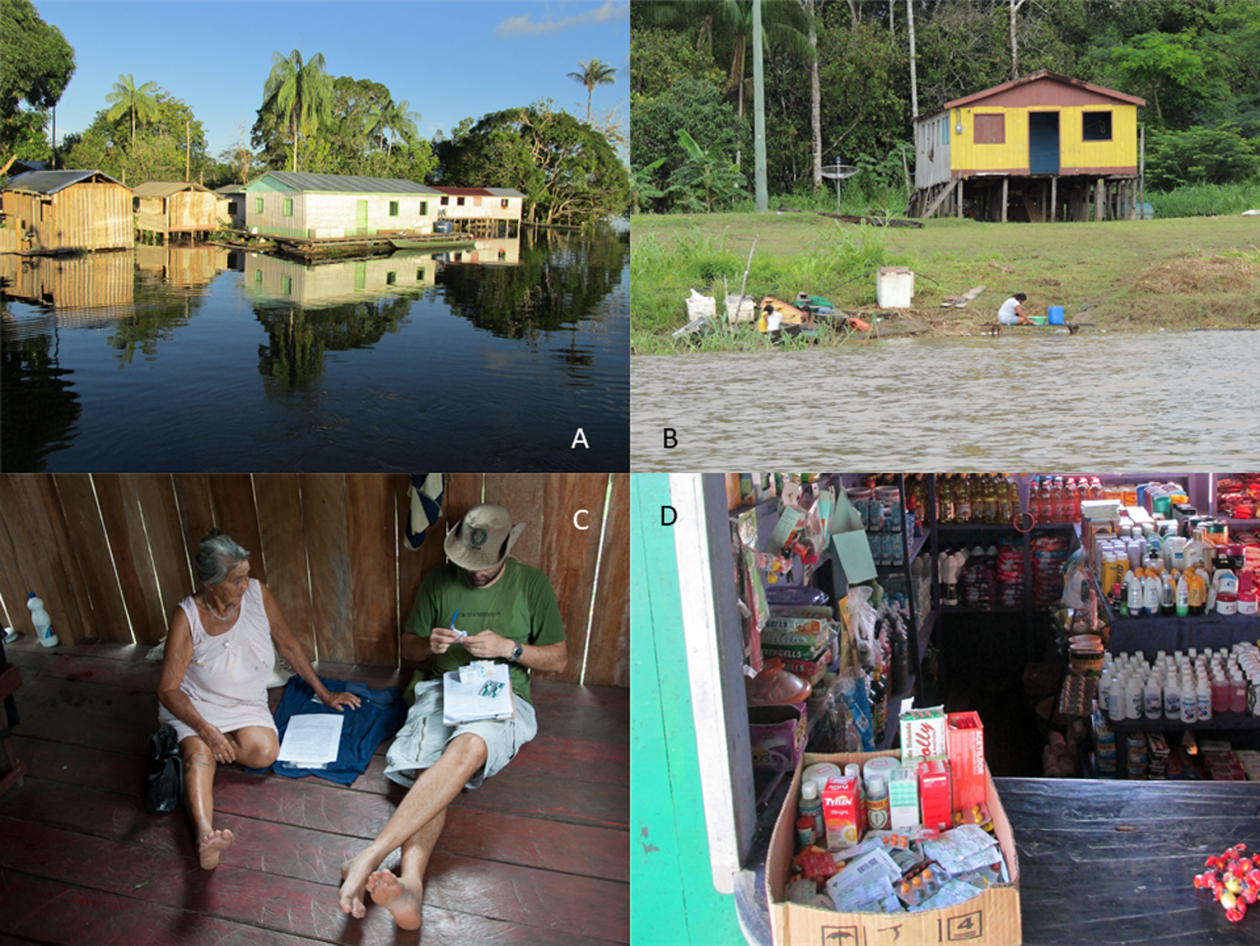
Photos illustrating typical life in the surveyed region. **(A)** Typical palaffitte houses of the river-dweller communities in the Amazonas region (*ribeirinhos*). These wooden houses are built over the land and float when water covers the area. (B) *Ribeirinhos* have easy access to fish, fluvial transportation and other activities. (C) Usual way to carry out the surveys in the riverside-dweller communities. The researcher (ASM-G) is examining the medication showed by the *ribeirinho* woman. (D) Small market in one of the riverside-dweller communities included in the study. Medicines aretypically sold in those shops.

The present survey was conducted in riverside-dwelling communities along the Amazonas River in Brazil. The photographs included in Fig 1 were taken by one of the researchers (AS M-G) in the location of the study (Coari) and show aspects of the riverside dwellers’ daily life. *Ribeirinhos* live in palaffites, wooden houses built over the land that float when the water level increases and floods the area. Far from urban areas, small markets sell many commodities, including medicines.

During the study, 492 *ribeirinhos* were interviewed (Table 1) and 346 (70.3%) consumed at least one medicine, either prescribed or as self-medication. A total of 74 patients (21.3% of those taking a medicine and 15.0% of the study sample) were using an AM.

**Table 1:** Main characteristics of the sample of riverside dwellers of Coari, Amazonas, Brazil, included in the study.

Variable		n (%)
Sex	male	231 (47.0)
	female	261 (53.0)
Age (years)	18–39	287 (58.3)
	40–59	149 (30.3)
	≥60	56 (11.4)
Distance to the closest urban area (km)	<50	253 (51.4)
	50 − 100	130 (28.3)
	>100	100 (20.3)
Time to reach the closest urban area (hours)	<1	54 (11.0)
	1 − 4	251 (51.0)
	>4	187 (38.0)
Frequency of travels to the closest urban zone	More than once per month	151 (30.7)
	Monthly	306 (62.2)
	Less than 1 trip per year	35 (7.1)

Analysis of the question about who recommended the AM showed that most were consumed without any medical prescription (49 patients, or 66.2% of those using AMs). In addition, as shown in Table 2, one out of three AMs consumed were used for a non-infectious or non-bacterial disease. Among the AMs consumed in self-medication were: amoxicillin where eight patients used it for “flu”, and “eye inflammation”; tetracycline six− patients used it to relieve “allergy”, “gut inflammation”, “flu”, and “joint inflammation”−, and sulfadiazine −six patients used it for “pain”, “flu”, and gut inflammation”−.

**Table 2:** AM use according to the prescription status and reason for consumption among the riverside dwellers of Coari, Amazonas, Brazil, 2016.

Antimicrobial	Prescribed	Non prescribed	Infectious conditions *	Non-infectious conditions**	Total n = 77 (%)
**Amoxicillin**	4	17	13	8	21 (27.3)
**Ampicillin**	3	8	8	3	11 (14.3)
**Cefalexin**	7	4	11	0	11 (14.3)
**Tetracycline**	0	10	4	6	10 (12.9)
**Sulfadiazine**	1	7	2	6	8 (10.4)
**Ciprofloxacin**	4	0	4	0	4 (5.2)
**Norfloxacin**	4	0	4	0	4 (5.2)
**Penicillin**	2	1	3	0	3 (3.9)
**Sulfamethoxazole**	0	2	1	1	2 (2.6)
**Azithromycin**	0	1	1	0	1 (1.3)
**Metronidazole**	1	0	0	1	1 (1.3)
**Methenamine**	0	1	1	0	1 (1.3)
**Total n (%)**	26 (33.8)	51 (66.2)	52 (67.5)	25 (32.5)	77 (100.0)

*Infectious conditions = tonsillitis, urinary tract infection, otitis, infected wound;

**Non-infectious/non-bacterial conditions = allergy, “gut inflammation” “pain” “flu” “eye inflammation” joint inflammation”.

Combined use of AMs without prescription was observed in three patients (4.1% of patients taking AMs). One patient was using ciprofloxacin and norfloxacin; another patient was concomitantly using ampicillin and penicillin, and the third was using amoxicillin with sulfadiazine.

## Discussion

Despite being forbidden in Brazil and many other countries, selling AMs without prescription is frequent even in remote places such as the Amazon Basin, which indicates both a risk of individuals suffering preventable adverse drug reactions and a risk of contributing to the worrying increase of AMR. In this survey of a representative sample of riverside dwellers, we found that 15% of the population had taken an AM, two-thirds of them without prescription and, even worse, in one-third of cases this was used to treat non-infectious or non-bacterial symptoms or conditions. Thus, these results show that AMs can be purchased without any restriction in pharmacies and even in small supermarkets, where over-the-counter and prescription medicines are sold in packages or by units. Among those medicines, analgesics and antimicrobial agents are the most commonly sold. The option of buying one blister or just “some pills” is especially relevant in the case of AMs, as this practice favors incomplete treatments, which is one of the well-known causes of AMR. On the other hand, and equally relevant, the use of AMs to treat health conditions that will not benefit from those medicines, because they are not caused by an infectious or bacterial agent, is an inappropriate practice and potentially dangerous both for the environment and for the patients who will not benefit from an AM and could suffer from adverse effects that might be even worse than their original disease. The use of amoxicillin or tetracycline for vague symptoms such as “gut inflammation”, for example, could be associated with subsequent AM−induced diarrhea. Likewise, indiscriminate use of tetracycline (12.9% of the 77 people exposed to an AM) in areas of bright sunshine and mostly outdoor living, presents a risk of phototoxic reactions.

The use of non-prescribed AMs and the use of AMs for non-infectious diseases are two worrying situations from a public health point of view. What should be highlighted from the present survey is that the findings were obtained from a very remote rural place, while most AM misuse has been described in urban areas, where an excessive number of AMs in the pharmaceutical market, aggressive pharmaceutical promotion, economic incentives where prescribers gain income from dispensing or selling the medicines they prescribe, and poor availability of independent pharmaceutical information such as clinical guidelines [15] have helped to increase the inappropriate use of medicines in general, and AM in particular.

Situations such as selling AMs without prescription and using them for non-infectious diseases clearly require a multifaceted approach addressed to pharmacists and pharmacy tenders, but also to the population. The use of AM stewardship programs has been recommended to address those situations [9], but this is complicated in places with poor or nonexisting healthcare structure such as the one studied herein. Regarding this point, community agents (*Agentes Comunitários de Saúde*, ACS) could be an important link for training in order to improve the basic health education of the riverside dwellers.

Table 1 shows some of the characteristics of the included population. Two interesting aspects are the distance and time needed to reach an urban area and frequency of travel to that urban nucleus. More than one-third of the studied population lived more than four hours away from the city and almost 70% went there only once per month or less. These figures give an idea of the isolation of the studied population.

There are some limitations inherent to the study design and the sample collection process. To reduce this, the interviews were carried out by a small team of researchers, and emphasis was put on trying not to influence the respondents. To obtain information regarding the use of medicines, riverside dwellers were asked to show the places where they were keeping medicines, the prescriptions and also the boxes or bottles, in order to aid identification and avoid mistakes. The precision of the description “non-infectious/non-bacterial” origin of the disease that prompted the consumption of a medicine should be carefully considered; for example, “gut inflammation” could be the lay expression used for “bloody diarrhea”. Notwithstanding this, the main objective of this analysis was to highlight the conditions of use of antimicrobials in remote places with poor access to healthcare systems and also to focus on the need to train community agents for a more rational use of medicines, especially antibiotics.

A qualitative approach could provide additional information about the knowledge and attitudes of the investigated population about the use of antibiotics. The results of the present quantitative approach will be used to inform a subsequent, larger research study analyzing attitudes and behaviors together with a precise map of the situation in the Amazon Basin, which will enable an an educational intervention addressed to users, healthcare teams, and health community agents.

Despite the potential limitations in the study design mainly attributable to the remote location of the Coari region, our results highlight the need for a higher level of control of the marketing of the AM medicines. In addition, the observations showed the unique reality of the studied region, which needs specific public health policies regarding the appropriate use of AMs. These actions will benefit riverside-dweller patients, and also should help to prevent unnecessary use of AMR and curb its environmental impact, as these populations are living along one of the most important rivers on Earth.

## Supporting information

S1 File Data collection sheet. Original version of the data collection sheet in Portuguese.
(PDF)

S2 File Data collection sheet. English version of the data collection sheet.
(PDF)
