# Supplementary material for: Inappropriately prescribed and over-the-counter antimicrobials in the Brazilian Amazon Basin: We need to promote more rational use even in remote places
Source: PLoS One. 2018 Aug 3;13(8):e0201579. doi: 10.1371/journal.pone.0201579 (PMC6075761; doi:10.1371/journal.pone.0201579)
Supplement: S1 File — Original version of the data collection sheet in Portuguese. (PDF) [file pone.0201579.s001.pdf]

Entrevistador: ____ **N. do Questionário:** I__I__I__I
Tempo/Entrevista: I__I__I__I **min** **Data:** ___/___/_____

INFORMAÇÕES AO ENTREVISTADO:

Antes de começar, gostaria de lhe agradecer por participar da pesquisa e informar que a entrevista é voluntária. Caso o Sr. (a) não deseje responder alguma pergunta, por favor me avise, pois passaremos para a próxima.

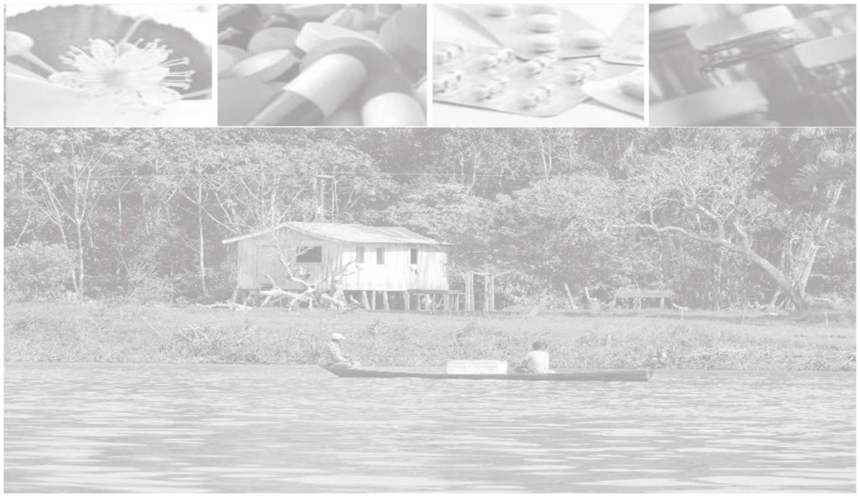

AUTOMEDICAÇÃO EM COMUNIDADES RIBEIRINHAS NA REGIÃO DO MÉDIO SOLIMÕES – AMAZONAS

UNIVERSIDADE DE SÃO PAULO – ESCOLA DE ENFERMAGEM

ENFERMAGEM NA SAÚDE DO ADULTO

UNIVERSIDADE FEDERAL DO AMAZONAS

UFAM

➢ ***INFORMAÇÕES GERAIS SOBRE O ENTREVISTADO E COMUNIDADE***

1 - Entrevistado: ____
2 - Comunidade: ________________________________ **3 - Região:** __________________________
4 - ACS da Comunidade: ____
5 - Origem: **S** ____º___’____” **W** ____º___’____” **6 - Domicílio: S** ____º___’____” **W** ____º___’____”
7 - N. do domicílio: I__I__I__I **8 - Indivíduo:** I__I__I__I **– Sua comunidade tem:**
9. Transporte comunitário? a. **S** ☐ b. **N** ☐ **10. Posto de saúde?** a. **S** ☐ b. **N** ☐
11. Telefone público/orelhão? a. **S** ☐ b. **N** ☐

Observações: ____
____
____

SEÇÃO A
"INFORMAÇÕES SÓCIO-DEMOGRÁFICOS"

A.1 – Qual a data de seu nascimento?__/__/___
"Não sabe informar, vá para A.2"

A.2 – Quantos anos o (a) Sr. (a) tem? I___I___I

A.3 – Sexo: a. M b. F

A.4 – O (a) Sr. (a) tem filhos? a. **S** b. **N**
"Não, vá para A.6"

A.5 - Quantos filhos nascidos vivos: I___I___I

A.6 – O (a) Sr. (a) nasceu nesta comunidade?
a. **S** b. **N** **"Sim, vá para A.8"**

A.7 – Onde o (a) Sr. (a) morava anteriormente?
____

A.8 - Há quanto tempo o (a) Sr. (a) mora nesta comunidade? a. I__I__I anos e I__I__I meses

A.9 – O (a) Sr. (a) já frequentou a escola?
a. **S** b. **N** **"Não, vá para A.11"**

A.10 - Até qual série? _____________________

A.11 – O (a) Sr. (a) sabe ler e escrever?
a. **S** b. **N**

A.12 – Contando com o (a) Sr. (a), quantas pessoas moram em sua casa?
a. número I__I__I
– Informações sobre os moradores:

Ord.	Idade	Sexo	Grau de parentesco
1			
2			
3			
4			
5			
6			
7			
8			
9			
10			
11			
12			
13			
14			
15			

A.13 - Qual é a sua religião?
a. Católico b. Evangélico c. Espírita
d. Nenhuma e. Outra:_________________

A.14 - Qual o seu estado marital hoje?
a. Solteiro b. Separado c. Viúvo
d. Casado/Civil e. Casado/Religioso
f. Casado/Civil/Religioso g. Mora junto

A.15 – O (a) Sr. (a) considera a sua cor da pele:
a. Branca b. Parda/Moreno (a)
c. Amarela d. Negra ou preta e. Indígena

A.16 – Incluindo a sua renda, quanto é aproximadamente a renda de todas as pessoas que moram com o (a) Sr. (a)? R$____________

A.17 – Com o que o (a) Sr. (a) trabalha ou faz durante o dia? ___________________________

A.18 - Na sua casa ou comunidade tem energia elétrica? a. **S** b. **N** **"Não, vá para A.20"**

A.19 - Qual a fonte de geração?
a. Motor b. Amaz. Energia c. E. solar

A.20 – Na sua casa tem:
a. Rádio: a. **S** b. **N** b. TV: a. **S** b. **N**
c. Celular: a. **S** b. **N** d. R. tran: a. **S** b. **N**

A.21 - Qual a origem da água que o (a) Sr. (a) usa para **cozinhar**?
a. Rio b. Igarapé c. Poço d. Chuva
e. Lago f. Outro **:____________________**

A.22 - A água para **cozinhar** é tratada?
a. **S** b. **N** - Se sim, qual o produto?____________

A.23 - Qual a origem da água que o (a) Sr. (a) usa para **beber**?
a. Rio b. Igarapé c. Poço d. Chuva
e. Lago f. Outro **:____________________**

A.24 - A água para **beber** é tratada?
a. **S** b. **N** - Se sim, qual o produto?____________

A.25 - Quantos cômodos tem sua casa? I__I__I

A.26 - Quando o rio enche, entra água na sua casa? a. **S** b. **N**

A.27 - Como o (a) Sr. (a) faz para chegar até Coari, o transporte é?
a. Alugado b. Cedido c. Próprio
d. Recreio/Pago e. Comunitário/público

A.28 - Quanto tempo o (a) Sr. (a) demora para chegar até Coari, com o transporte que usa normalmente? I__I__I horas e I__I__I minutos

A.29 - De quanto em quanto tempo o (a) Sr. (a) costuma ir em Coari?
a. Todos dias b. Até 3 vezes por semana
c. 1 vez por semana d. 1 vez por quinzena
e. 1 vez por mês f. Não costumo ir
- Se **"f"**, quanto tempo faz desde a última vez?
I__I__I anos I__I__I meses

SEÇÃO B
"ACESSO AOS SERVIÇOS DE SAÚDE"

B.1 – Quando o (a) Sr. (a) fica doente, quem costuma procurar primeiro?
a. Médico b. Enfermeiro c. Dentista
d. Farmacêutico e. ACS f. Rezador
g. Amigo, vizinho ou conhecido h. Pegador
i. Curandeiro j. Não procuro
k. Outro ______________________________

B.2 – Qual serviço de saúde o (a) Sr. (a) prefere utilizar quando está doente?
a. Posto de Saúde/Centro b. HRC
c. IMTC d. Outro Posto de Saúde
e. Policlínica f. Clínica/particular
g. Farmácia h. Nunca fui/serviço de saúde
i. Outro **:**______________________________

B.3 – No último mês, o (a) Sr. (a) procurou algum serviço de saúde para cuidar de sua saúde?
a. S **b. N** **"Não, vá para B.8"**

B.4 - Quanto tempo o (a) Sr. (a) levou para procurar o serviço de saúde? _______________

B.5 - O (a) Sr. (a) resolveu seu problema de saúde? **S** **N** **"Sim, vá para B.8"**

B.6 – Por que não foi resolvido?
____
____

B.7 – O que fez para resolver o seu problema?
____
____

B.8 - Como o (a) Sr. (a) costuma chegar até o serviço de saúde?
a. Lancha da comunidade b.Rabeta
c.Canoa a remo d.Recreio/pago d. Barco
e. A pé f. Moto g. Caminhão
h. Outro **:**______________________________

B.9 - Quanto tempo o (a) Sr. (a) costuma levar para chegar ao serviço de saúde quando precisa? I___I___I horas e I___I___I minutos

B.10 – O (a) Sr. (a) já precisou fazer acompanhamento de alguma doença?
a. **S** b. **N** **"Não, vá para B.12"**

B.11 – Teve alguma dificuldade?
a. **S** b. **N**
c. Se "sim", qual a dificuldade?
____
____

B.12 – Quando o (a) Sr. (a) está doente, e o profissional de saúde marca um exame, o (a) Sr. (a) consegue fazer?
a. **S** b. **N** c. Nunca fiz exame
"Sim, vá para B.14"

B.13 – Qual a dificuldade em fazer o exame?
____
____

B.14 – O (a) Sr. (a) já tentou tirar uma ficha ou marcar uma consulta e não conseguiu?
a. **S** b. **N** c. Nunca consultei
"Não, vá para B.16"

B.15 – Por que não conseguiu?
____
____

B.16 – Se um profissional de saúde, passa um remédio para o (a) Sr. (a) tomar, você consegue o remédio?
a. **S** b. **N** c. Nunca passou remédio
"Sim, pule para próxima seção"

B.17 – Qual o motivo de não conseguir o remédio?
____
____

SEÇÃO C
"AUDIT ADAPTADO - RIBEIRINHOS"

C.1 - Com que frequência o (a) Sr. (a) consome bebidas alcoólicas?
(0) Nunca ☐ **"NUNCA, vá próxima seção"**
(1) Mensalmente ou menos ☐
(2) De 2 a 4 vezes por mês ☐
(3) De 2 a 3 vezes por semana ☐
(4) 4 ou mais vezes por semana ☐

C.2 – Nas ocasiões em que bebe, o que bebe? I________________________I. Quantas doses o (a) Sr. (a)? **(Ver próxima página, ilustração sobre as doses - AUDIT)**
(0) 0 ou 1 ☐
(1) 2 ou 3 ☐
(2) 4 ou 5 ☐
(3) 6 ou 7 ☐
(4) 8 ou mais ☐

C.3 - Quantas vezes o (a) Sr. (a) toma 6 ou mais doses em uma mesma ocasião?
(0) Nunca ☐
(1) Menos do que uma vez ao mês ☐
(2) Mensalmente ☐
(3) Semanalmente ☐
(4) Todos ou quase todos os dias
"Se a soma das questões C.2 e C.3 for 0, avance para as questões C.9 e C.10"

C.4 – Durante o último ano, quantas vezes o (a) Sr. (a) achou que não conseguiria parar de beber depois de ter começado?
(0) Nunca ☐
(1) Menos que uma vez ao mês ☐
(2) Mensalmente ☐
(3) Semanalmente ☐
(4) Todos ou quase todos os dias ☐

C.5 – Durante o último ano, quantas vezes depois de ter bebido, o (a) Sr. (a) deixou de fazer alguma coisa que normalmente faria?
(0) Nunca ☐
(1) Menos que uma vez ao mês ☐
(2) Mensalmente ☐
(3) Semanalmente ☐
(4) Todos ou quase todos os dias ☐

C.6 – Durante o último ano, quantas vezes o (a) Sr. (a) precisou beber pela manhã para se sentir bem depois ter bebido muito/pesadamente no dia ou na noite anterior?
(0) Nunca ☐
(1) Menos que uma vez ao mês ☐
(2) Mensalmente ☐
(3) Semanalmente ☐
(4) Todos ou quase todos os dias ☐

C.7 – Durante o último ano, quantas vezes o (a) Sr. (a) se sentiu culpado ou com remorso depois de ter bebido?
(0) Nunca ☐
(1) Menos que uma vez ao mês ☐
(2) Mensalmente ☐
(3) Semanalmente ☐
(4) Todos ou quase todos os dias ☐

C.8 - Durante o último ano, quantas vezes o (a) Sr. (a) não foi capaz de lembrar o que aconteceu depois de ter bebido na noite anterior?
(0) Nunca ☐
(1) Menos que uma vez ao mês ☐
(2) Mensalmente ☐
(3) Semanalmente ☐
(4) Todos ou quase todos os dias ☐

C.9 - Já aconteceu do (a) Sr. (a) ferir ou magoar alguém ou de te ferir ou te magoar por causa de ter bebido?
(0) Não ☐
(2) Sim, mas não nos últimos 12 meses ☐
(4) Sim, nos últimos 12 meses ☐

C.10 - Algum parente, amigo, médico ou outro profissional já ficou preocupado com a forma que (a) Sr. (a) bebe ou sugeriu que diminuísse?
(0) Não
(2) Sim, mas não nos últimos 12 meses
(4) Sim, nos últimos 12 meses

SEÇÃO D
"HÁBITO TABAGISTA"

D.1 – O (a) Sr. (a) é ou já foi fumante (pelo menos 100 cigarros ao longo da vida)? a. **S** ☐
D.1.1 – O que costuma (va) fumar?
____
b. **N** ☐ **"Não, vá para seção seguinte"**

D.2 – Com que idade o (a) Sr. (a) começou a fumar? I__I__I anos

D.3 – O (a) Sr. (a) fuma cigarros atualmente?
a. **S** ☐ (**Sim, vá para D.5**) b. **N** ☐

D.4 – Com que idade o (a) Sr. (a) parou de fumar pela última vez? I__I__I anos

D.5 – Em geral, quantos cigarros por dia o (a) Sr. (a) fuma (ou fumava)?
I__I__I cigarros (se <1, marque 0)

D.6 – Ao todo, durante quantos anos o (a) Sr. (a) fumou ou fuma?
I__I__I anos (se <1, marque 0)

SEÇÃO E
“ENFERMIDADES AUTO-RELATADAS”

E.1 – O (a) Sr. (a) diria que sua saúde é?
a. Muito boa □ b. Boa □ c. Regular □
d. Ruim □ e. Muito ruim □

E.2 – No último mês, o (a) Sr. (a) teve algum problema de saúde, ou um profissional de saúde lhe disse que o (a) Sr. (a) tinha algum problema?
a. **S** □ b. **N** □
<u>“Não, vá para E.4</u>

E.3 - Qual foi o problema de saúde?

	**Enfermidades**	**Último mês**
**a**	Malária	□
**b**	Verminoses	□
**c**	Doença de Chagas	□
**d**	Hepatite	□
**e**	Diarreia	□
**f**	Pressão alta	□
**g**	Diabetes	□
**h**	Derrame	□
**i**	Câncer	□
**j**	Reumatismo	□
**k**	Doença no coração	□
**l**	Doença nos rins	□
**m**	Doenças nos olhos	
**n**	Alergia	□
**o**	Asma	□
**p**	Anemia	□
**q**	Gripe	□
**r**	Doenças pulmonares	□
**s**	Infecção urinária	□
**t**	Amidalite	
**u**	Fraqueza/vertigem/tontura	□
**v**	Azia (gastura)	□
**x**	Vômitos (provocou)	□
**z**	Falta de ar	□
**w**	Cansa facilmente	□
**aa**	Pressão na cabeça	□
**ab**	Febre	□
**ac**	Torção (desmintidura)	□
**ad**	Doença mental	□
**ae**	Depressão	□
Outros, especifique abaixo:		
**af**		□
**ag**		□
**ah**		□
**ai**		□
**aj**		□
**ak**		□
**al**	Dor (especificar local da dor):	□

E.4 - Alguma vez o (a) Sr. (a) sentiu dor ou desconforto no peito?
a. **S** □ b. **N** □ **<u>“Não” vá para seção seguinte”</u>**

E.5 - Esta dor ou desconforto aparece quando o (a) Sr. (a) caminha rápido ou trabalha no roçado?
a. **S** □ b. **N** □

E.6 - Esta dor ou desconforto aparece quando o (a) Sr. (a) caminha devagar em terreno plano?
a. **S** □ b. **N** □

“ILUSTRAÇÃO AUDIT”

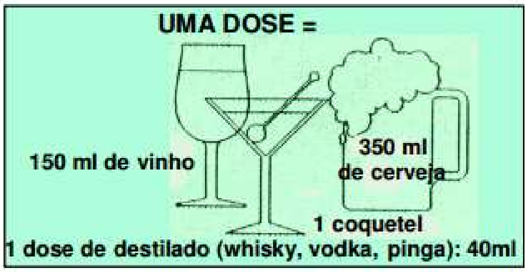

SEÇÃO F
"INFORMAÇÕES SOBRE O CONSUMO DE MEDICAMENTOS"

F.1 – O (a) Sr. (a) usa "remédios de farmácia" por conta própria? a. **S** ☐ b. **N** ☐

F.2 – O (a) Sr. (a) guarda algum "remédio de farmácia" em casa? a. **S** ☐ b. **N** ☐
"Não, vá para E.5"

F.3 - Onde guarda seus "remédios de farmácia" em casa (peça para mostrar o local)?
____
____
____

⇨ SE POSSÍVEL, PEÇA A CAIXINHA DE MEDICAMENTOS.

F.4 – Características do local onde os "remédios de farmácia" são guardados na residência **(observação do entrevistador)**?
a. Contato direto dos raios solares ☐
b. Contato com umidade ☐
c. Contato direto com calor ☐
d. Outras observações do entrevistador:
____
____
____

F.5 - Como o (a) Sr. (a) identifica seus "remédios de farmácia"?
a.Pela cor ☐ b. Pelo tamanho ☐ c. Pela forma☐
d. Pelo nome ☐ e. Pela embalagem ☐
f. Não identifica ☐ g. Outro ☐**:**______________

F.6 - De um modo geral o (a) Sr. (a) costuma ler a bula dos remédios ou pede para alguém ler?
a. **S** ☐ b. **N** ☐

F.7 - O (a) Sr. (a) acha que os "remédios de farmácia" podem fazer mal a sua saúde?
a. **S** ☐ b. **N** ☐

F.8 - O (a) Sr. (a) costuma ver a validade de seus "remédios de farmácia"?
a. **S** ☐ b. **N** ☐

F.9 - O (a) Sr. (a) já tomou algum "remédio de farmácia" vencido?
a. **S** ☐ b. **N** ☐ c. **Não sei informar** ☐

F.10 – Quando o "remédio de farmácia" que o (a) Sr. (a) tem em sua casa, vence, onde costuma jogar seus remédios?
____
____

F.11 - O (a) Sr. (a) costuma guardar o "remédio de farmácia" dentro das caixinhas **(embalagem secundária)**? a. **S** ☐ b. **N** ☐

F.12 - O (a) Sr. (a) costuma retirar os comprimidos da cartela dos "remédios de farmácia" para guardar em outro local?
a. **S** ☐ b. **N** ☐

F.13 – O (a) Sr. (a) costuma aproveitar os potes de "remédios de farmácia"?
a. **S** ☐ b. **N** ☐

F.14 – O (a) Sr. (a) usou remédio caseiro no último mês?
a. **S** ☐ b. **N** ☐ **"Não, vá para F.18"**

F.15 – Qual remédio caseiro usou?
____
____

F.16 – Para que usou o remédio caseiro?
____
____

F.17 – Quem lhe ensinou usar?
____
____

F.18 – Quando o (a) Sr. (a) está doente, o que prefere usar:
a. Remédio caseiro ☐
b. Remédio de farmácia ☐
c. Remédio Caseiro e de farmácia ☐
d. Não uso nada ☐

F.19 – Por que?
____
____

F.20 - O (a) Sr. (a) tomou algum "remédio de farmácia" no último mês? **a. S** ☐ **b. N** ☐

"Entrevistado (a) não tomou remédio de farmácia no último mês, *VÁ PARA PRÓXIMA SEÇÃO"*

⇨ CASO O ENTREVISTADO TENHA TOMADO REMÉDIO DE FARMÁCIA NO ÚLTIMO MÊS, PEÇA PARA MOSTRAR A RECEITA MÉDICA (SE HOUVER) E O MEDICAMENTO.

⇨ PREENCHA O QUADRO ABAIXO COM AS INFORMAÇÕES EMITIDAS PELO ENTREVISTADO.

REMÉDIO DE FARMÁCIA (MEDICAMENTO ALOPÁTICO)					
Ord.	F.21	F.22	F.23	F.24	F.25
	Nome do "remédio de farmácia" (medicamento alopático)	Tempo de uso	Quem indicou?	Onde conseguiu o "remédio de farmácia"?	Para que usou?
a	______________________<br>______________________<br>______________________	Dias I__I__I<br>Meses I__I__I<br>Anos I__I__I	(a) Médico ☐ (b) Dentista ☐<br>(c) Farmacêutico ☐ (d) Enfermeira ☐<br>(e) ACS ☐ (f) Conta própria ☐<br>(g) Vizinho/amigo ☐ (h) Familiar ☐<br>(i) Outro ☐:__________________	(a) Farmácia ☐ (b) Posto de Saúde ☐<br>(c) Hospital ☐ (d) IMTC ☐ (e) Policlínica ☐<br>(f) ACS ☐ (g) Vizinho/amigo☐<br>(h) Familiar ☐ (i) Outro ☐:____________	______________________<br>______________________<br>______________________
b	______________________<br>______________________<br>______________________	Dias I__I__I<br>Meses I__I__I<br>Anos I__I__I	(a) Médico ☐ (b) Dentista ☐<br>(c) Farmacêutico ☐ (d) Enfermeira ☐<br>(e) ACS ☐ (f) Conta própria ☐<br>(g) Vizinho/amigo ☐ (h) Familiar ☐<br>(i) Outro ☐:__________________	(a) Farmácia ☐ (b) Posto de Saúde ☐<br>(c) Hospital ☐ (d) IMTC ☐ (e) Policlínica ☐<br>(f) ACS ☐ (g) Vizinho/amigo☐<br>(h) Familiar ☐ (i) Outro ☐:____________	______________________<br>______________________<br>______________________
c	______________________<br>______________________<br>______________________	Dias I__I__I<br>Meses I__I__I<br>Anos I__I__I	(a) Médico ☐ (b) Dentista ☐<br>(c) Farmacêutico ☐ (d) Enfermeira ☐<br>(e) ACS ☐ (f) Conta própria ☐<br>(g) Vizinho/amigo ☐ (h) Familiar ☐<br>(i) Outro ☐:__________________	(a) Farmácia ☐ (b) Posto de Saúde ☐<br>(c) Hospital ☐ (d) IMTC ☐ (e) Policlínica ☐<br>(f) ACS ☐ (g) Vizinho/amigo☐<br>(h) Familiar ☐ (i) Outro ☐:____________	______________________<br>______________________<br>______________________
d	______________________<br>______________________<br>______________________	Dias I__I__I<br>Meses I__I__I<br>Anos I__I__I	(a) Médico ☐ (b) Dentista ☐<br>(c) Farmacêutico ☐ (d) Enfermeira ☐<br>(e) ACS ☐ (f) Conta própria ☐<br>(g) Vizinho/amigo ☐ (h) Familiar ☐<br>(i) Outro ☐:__________________	(a) Farmácia ☐ (b) Posto de Saúde ☐<br>(c) Hospital ☐ (d) IMTC ☐ (e) Policlínica ☐<br>(f) ACS ☐ (g) Vizinho/amigo☐<br>(h) Familiar ☐ (i) Outro ☐:____________	______________________<br>______________________<br>______________________
e	______________________<br>______________________<br>______________________	Dias I__I__I<br>Meses I__I__I<br>Anos I__I__I	(a) Médico ☐ (b) Dentista ☐<br>(c) Farmacêutico ☐ (d) Enfermeira ☐<br>(e) ACS ☐ (f) Conta própria ☐<br>(g) Vizinho/amigo ☐ (h) Familiar ☐<br>(i) Outro ☐:__________________	(a) Farmácia ☐ (b) Posto de Saúde ☐<br>(c) Hospital ☐ (d) IMTC ☐ (e) Policlínica ☐<br>(f) ACS ☐ (g) Vizinho/amigo☐<br>(h) Familiar ☐ (i) Outro ☐:____________	______________________<br>______________________<br>______________________
f	______________________<br>______________________<br>______________________	Dias I__I__I<br>Meses I__I__I<br>Anos I__I__I	(a) Médico ☐ (b) Dentista ☐<br>(c) Farmacêutico ☐ (d) Enfermeira ☐<br>(e) ACS ☐ (f) Conta própria ☐<br>(g) Vizinho/amigo ☐ (h) Familiar ☐<br>(i) Outro ☐:__________________	(a) Farmácia ☐ (b) Posto de Saúde ☐<br>(c) Hospital ☐ (d) IMTC ☐ (e) Policlínica ☐<br>(f) ACS ☐ (g) Vizinho/amigo☐<br>(h) Familiar ☐ (i) Outro ☐:____________	______________________<br>______________________<br>______________________
g	______________________<br>______________________<br>______________________	Dias I__I__I<br>Meses I__I__I<br>Anos I__I__I	(a) Médico ☐ (b) Dentista ☐<br>(c) Farmacêutico ☐ (d) Enfermeira ☐<br>(e) ACS ☐ (f) Conta própria ☐<br>(g) Vizinho/amigo ☐ (h) Familiar ☐<br>(i) Outro ☐:__________________	(a) Farmácia ☐ (b) Posto de Saúde ☐<br>(c) Hospital ☐ (d) IMTC ☐ (e) Policlínica ☐<br>(f) ACS ☐ (g) Vizinho/amigo☐<br>(h) Familiar ☐ (i) Outro ☐:____________	______________________<br>______________________<br>______________________

SEÇÃO H
"DADOS NUTRICIONAIS"

H.1 - Quantas refeições o (a) Sr. (a) faz ao dia (considere café da manhã, almoço, jantar e merendas)? ___
H.2 - Quantas vezes por semana você come os alimentos abaixo?

**Ord.**	**ALIMENTOS**	Nunca quase nunca	1x/ mês	2 x/ mês	1x/ sem	2-3 x/ sem	4-5x/ sem	Todos os dias
**1**	Carne vermelha (boi)							
**2**	Carne vermelha (porco)							
**3**	Carne vermelha (caça)							
**4**	Carne de aves (Frango, galinha, pato)							
**5**	Peixes							
**6**	Ovos							
**7**	Feijão							
**8**	Arroz ou macarrão							
**9**	Farinhas e pães (mandioca, beiju, rosca)							
**10**	Leite							
**11**	Verduras de folhas (alface, couve, etc)							
**12**	Legumes (cenoura, jerimum, beterraba, etc)							
**13**	Legumes tubérculos (macaxeira, cará, batata)							
**14**	Frutas ou suco de fruta natural							
**15**	Açaí							
**16**	Pupunha							
**17**	Tucumã							
**18**	Guaraná natural							
**19**	Castanha							
**20**	Açúcar							
**21**	Doces (bombons, chiclete, pirulito)							
**22**	Produtos industrializados (carne enlatada, salsicha enlatada, salgadinhos, etc)							

SEÇÃO I
"SCREENING PARA FRAGILIDADE"
(Idosos > de 60 anos)

I.1 - Nos últimos 12 meses, o (a) Sr. (a) se sente mais enfraquecido (a), acha que sua força diminuiu? a. **S** b. **N**

I.2 - Atualmente, o (a) Sr. (a) faz menos atividades físicas do que fazia há 12 meses atrás (há um ano)? a. **S** b. **N**

I.3 - Atualmente, o (a) Sr. (a) acredita que está caminhando mais devagar do que há 12 meses atrás (há um ano / demora mais tempo para fazer o mesmo percurso que fazia antes, como ir à igreja, na cidade, etc)? a. **S** b. **N**

I.4 - Com que frequência, na última semana, o (a) Sr. (a) sentiu que não conseguiria levar adiante suas coisas (iniciava alguma coisa mas não conseguia terminar):
a. Nunca ou raramente (< de 1 dia)
b. Poucas vezes (1-2dias) c. Algumas vezes (3-4 dias)
d. A maior parte de tempo

I.5 - Com que frequência, na última semana, a realização de suas atividades rotineiras exigiram do (a) Sr. (a) um grande esforço para serem realizadas:
a. Nunca ou raramente (< de 1 dia)
b. Poucas vezes (1-2 dias) c. Algumas vezes (3-4 dias)
d. A maior parte de tempo

SEÇÃO J
"DADOS ANTROPOMÉTRICOS, PRESSÃO ARTERIAL, GLICEMIA CAPILAR E TIPAGEM SANGUÍNEA"

J.1 – Pressão arterial:
a. (1)___x____mmHg b. (2) ___x____mmHg
c. (3)____x____mmHg
J.2 - Impossibilitado de realizar medidas antropométricas: Motivo? _________________
J.3 - Estatura ______cm **J.4 –** Peso____ kg
J.5 - Circunferência da cintura ___________ cm
J.6 – C. quadril ___ cm **J.7 –** C. braço ___ cm
J.8 – C. panturrilha _____ cm **J.9 -** FC:___bpm
J.10 - Há quanto tempo o (a) Sr. (a) se alimentou a última vez? I__I__I horas e I__I__I minutos
J.11 – O que o (a) Sr. (a) comeu na sua última refeição? ___________________________________
____
J.12 – Glicemia capilar_________
J.13 – Você sabe qual o seu tipo sanguíneo?
a. **S** b. **N** **c.** Qual? _______________
J.14 – Tipo sanguíneo: ABO I___I Rh I___I
