# Supplementary material for: Inappropriately prescribed and over-the-counter antimicrobials in the Brazilian Amazon Basin: We need to promote more rational use even in remote places
Source: PLoS One. 2018 Aug 3;13(8):e0201579. doi: 10.1371/journal.pone.0201579 (PMC6075761; doi:10.1371/journal.pone.0201579)
Supplement: S2 File — English version of the data collection sheet. (PDF) [file pone.0201579.s002.pdf]

**Interviewer:** ____ **Survey Nr:** I__I__I__I<br>**Survey time:** I__I__I__I **min** **Date:** ___/___/_____

INFORMATIONS FOR THE INTERVIEWEE

First of all, I would like to express our gratitude for your participation in this survey. This is a voluntary survey; in the case you prefer not to take part of it, please tel me. If you prefer not to answer a specific question, please tell me too.

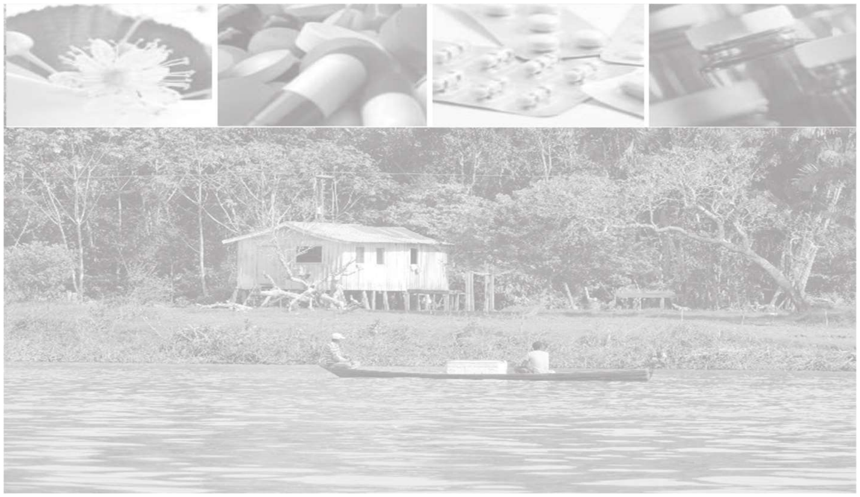

SELFMEDICATION IN RIVER DWELLER COMMUNITIED OF THE MÉDIO SOLIMÕES REGION – AMAZONAS

UNIVERSIDADE DE SÃO PAULO – ESCOLA DE ENFERMAGEM

ENFERMAGEM NA SAÚDE DO ADULTO

UNIVERSIDADE FEDERAL DO AMAZONAS

UFAM

- ***GENERAL INFORMATION ABOUT THE COMMUNITY***

1 - Interviewee: ____

2 - Community: ________________________________ **3 - Region:** __________________________

4 – Community ACS: ____

5 - Origin: **S** ____º___’____” **W** ____º___’____” **6 - Address: S** ____º___’____” **W** ____º___’____”

7 – Hose Nr: I__I__I__I **8 – Person Nr:** I__I__I__I **– The community has:**

9. Public transportation? a. **Y** ☐ b. **N** ☐ **10. Health Center?** a. **Y** ☐ b. **N** ☐

11. Public phone? a. **Y** ☐ b. **N** ☐

Observations: ____

____

____

SECTION A
"SOCIODEMOGRAPHIC INFORMATION"

A.1 – Birthdate?__/__/___
"Do not know", go to A.2

A.2 – How old are you? I___I___I

A.3 – Sex: a. M b. F

A.4 – Family? a. **Y** b. **N**
"No", go to A.6

A.5 – How many live children?: I___I___I

A.6 – Have you born in this community?
a. **Y** b. **N** **"Yes", go to A.8"**

A.7 – Where did you live before?
____

A.8 – When did you arrive at the community? a. I__I__I Year I__I__I Month

A.9 – Did you go to the school?
a. **Y** b. **N** **"No", go to A.11**

A.10 – Last degree? _____________________

A.11 – Can you read/write?
a. **Y** b. **N**

A.12 – How many people are leaving in this house?
a. Nr I__I__I
– Informations on the residents:

Ord.	Age	Sex	Kinship
1			
2			
3			
4			
5			
6			
7			
8			
9			
10			
11			
12			
13			
14			
15			

A.13 - Religion?
a. Catholic b. Evangelic c. Spirit
d. None e. Other:_________________

A.14 – Marital status?
a. Single b. Divorced c. Widow
d. Married/ civil e. Married/religious
f. Married/Both g. Living together

A.15 – Skin color:
a. White b. Swarthy
c. Yellow d. Black e. Indigenous

A.16 – Total home income? R$____________

A.17 – Occupation?
____

A.18 – Do you have electricity? a. **Y** b. **N**
"No", go to A.20

A.19 – Power source?
a. Engine b. Battery c. Solar

A.20 – Do you have:
a. Radio: a. **Y** b. **N** b. TV: a. **Y** b. **N**
c. Phone: a. **Y** b. **N** d. R. transm: a. **Y** b. **N**

A.21 – Water source / **cooking**?
a. Rivero b. *Igarapé* c. Well d. Rain
e. Lake f. Other **:____________________**

A.22 – Is **cooking** water treated??
a. **Y** b. **N** - If Yes, product name?___________

A.23 – Water source **/ drinking**?
a. River b. Igarapé c. Well d. Rain
e. Lake f. Other **:____________________**

A.24 – Is **drinking** water treated?
a. **Y** b. **N** - If Yes, product name?___________

A.25 – Number of rooms? I__I__I

A.26 – When the river rises, your home is flooded? a. **Y** b. **N**

A.27 – You reach Coari by transportation?
a. Rented b. Lend c. Own
d. Paid e. Public

A.28 – How long does it take to reach Coari? I__I__I hours I__I__I minutes

A.29 – When do you use to go to Coari?
a. Daily b. 3 times/week c. 1 time/week
 d. 2 times/month
e. 1 time/month f. Do not use to go
- If **"f"**, when did you go last time? I__I__I years I__I__I months

SECTION B
"HEATH CARE ACCESS"

B.1 – When you get sick, who do you seek first?
a. Physician b. Nurse c. Dentist
d. Pharmacist e. CHA f. Preacher
g. Friend h. Neighbour i. Healer
j. Nobody k. Other ___________________

B.2 – Healthcare servisse of your first choice?
a. Aid station b. HRC
c. IMTC d. Other
e. Policlinic f. Private clinic
g. Pharmacy h. Never went to a healthcare center i. Other:____________________

B.3 – In the last month, have you gone to a healthcentre? **a. Y** **b. N**
"No", go to B.8"

B.4 – How long did it take to reach the healthcentre? ________________

B.5 – Is your health problem solved? **Y** **N**
"Yes", go to B.8

B.6 – Why haven't it been solved?
____
____

B.7 – What did you do to solve the problem?
____
____

B.8 – How do you reach the healthcare c.?
a. Community boat b.*Rabeta* c.Oar canoe d.Paying boat d. Ship e. Walking
f. Motorcycle g. Truck
h. Other :_______________________________

B.9 - Usual time to reach the healthcare center?
I___I___I hours I___I___I minutes

B.10 – Did you need any disease follow-up in the past?
a. **Y** b. **N** **"No", go to B.12**

B.11 – Had any difficulty?
a. **Y** b. **N**
c. If "yes", which one?
____
____

B.12 – When you required a lab test, was it done?
a. **Y** b. **N** c. Never had a test
"Yes", go to B.14

B.13 – Which problem did you found to get the test?
____
____

B.14 – Did you ever need a consultation and could'nt do it?
a. **Y** b. **N** c. Never need a consultation
"No", go to B.16

B.15 – Why didn't you get it?
____
____

B.16 – If a a health professional prescribes you a remedy, do you use to get it?
a. **Y** b. **N** c. Never needed one
"Yes", go to next section"

B.17 – Why did you not get it?
____
____

SETION C
"ADAPTED AUDIT - RIBEIRINHOS"

C.1 – Alcoholic beverages?
(0) Nunca □ **"NUNCA, vá próxima seção"**
(1) Monthly □
(2) 2 - 4 times / month □
(3) 2 - 3 times / week □
(4) 4 or more times/week □

C.2 – When you drink, what do you drink? I________________________I. Dosages? **(See next page Figure)**
(0) 0 - 1 □
(1) 2 - 3 □
(2) 4 - 5 □
(3) 6 - 7 □
(4) 8 or more □

C.3 – How many time do you use to have 6 or more doses?
(0) Never □
(1) Less than 1/month □
(2) Monthly □
(3) Weekly □
(4) Daily
"If adding C.2 and C.3 equals 0, go to C.9 and C.10"

C.4 – During the last year, how many time did you think that you will manage to stop drinking?
(0) Never □
(1) Less than 1/month □
(2) Monthly □
(3) Weekly □
(4) Almost daily or daily □

C.5 – During the last year, how many times you could not do anything because you had been drinking?
(0) Never □
(1) Less than 1/month □
(2) Monthly □
(3) weekly □
(4) Almost daily or daily □

C.6 – During the last year, how many times did you feel the need to drink in order to improve after having been drinking the night before?
(0) Never □
(1) Less than 1/month □
(2) Monthly □
(3) Weekly □
(4) Almost daily or daily □

C.7 – During the last year, how many times did you feel remorse for having been drinking?
(0) Never □
(1) Less than 1/month □
(2) Monthly □
(3) Weekly □
(4) Almost daily or daily □

C.8 – During the last year, how many times you were not able to remember what happened after being drinking the night before?
(0) Never □
(1) Less than 1/month □
(2) Monthly □
(3) weekly □
(4) Almost daily or daily □

C.9 – Have you ever hurt or wounded anyone after having been drinking?
(0) No □
(2) Yes, but not during the last year □
(4) Yes, during the last year □

C.10 – Has any Family member por friend been worried for you or suggested to drink less?
(0) No
(2) Yes, but not during the last year
(4) Yes, during the last year

SECTION D
"SMOKING HABIT"

D.1 – Have you been a smoker (at least 100 cigarettes during your life)? a. **Y** □
D.1.1 – What do you use to smoke?

____
b. **N** □ **"No", go to the next Section**

D.2 – When did you begin to smoke?
I__I__I years

D.3 – Do you smoke cigarettes?
a. **Y** □ (**Yes, go to D.5**) b. **N** □

D.4 – When did you quit the smoking habit?
I__I__I years

D.5 – How many cigarettes/day do or did you smoke?
I__I__I cigarettes (if <1, write 0)

D.6 – How many years have you been smoking?
I__I__I years (if <1, write 0)

SECTION E
"SELF-REPORTED DISEASES"

E.1 – Your health is?
a. Very good □ b. Good □ c. Fair □
d. Bad □ e. Very bad □

E.2 – During the last month, did you presente any health problem? a. **Y** □ b. **N** □
"No", go to E.4

E.3 – Which was your health problem?

	**Diseases**	**Last month**
**a**	Malaria	□
**b**	Parasitoses	□
**c**	Chagas disease	□
**d**	Hepatitis	□
**e**	Diarrhea	□
**f**	Hjigh blood pressure	□
**g**	Diabetes	□
**h**	Hemorrhage	□
**i**	Cancer	□
**j**	Rheumatism	□
**k**	Heart disease	□
**l**	Kidney disease	□
**m**	Eye disease	
**n**	Allergy	□
**o**	Asthma	□
**p**	Anemia	□
**q**	Flu	□
**r**	Lung disease	□
**s**	Urinary infection	□
**t**	Tonsilitis	
**u**	Dizziness / vertigo	□
**v**	Heartburn	□
**x**	Vomiting	□
**z**	Short breath	□
**w**	Feeling weak	□
**aa**	Head pressure	□
**ab**	Fever	□
**ac**	Colic/cramps	□
**ad**	Mental simptoms	□
**ae**	Depression	□
Other, please, detail:		
**af**		□
**ag**		□
**ah**		□
**ai**		□
**aj**		□
**ak**		□
**al**	Pain (please specify):	□

E.4 – Have you ever felt pain or discomfort on the heart zone?
a. **Y** □ b. **N** □ **"No" go to the next Section"**

E.5 – This discomfort uses to appear when you are walking quickly or are working?
a. **Y** □ b. **N** □

E.6 – This discomfort uses to appear when you are walking slowly? a. **Y** □ b. **N** □

"FIGURE AUDIT"

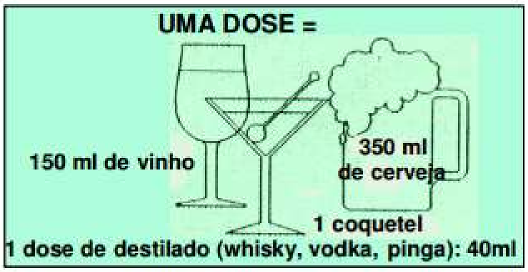

SETION F
"INFORMATIONS ON MEDICINES CONSUMPTION"

F.1 – Do you use "pharmacy remedies" on your own? a. **Y** ☐ b. **N** ☐

F.2 – Do you keep any "pharmacy remedy" at home? a. **Y** ☐ b. **N** ☐
<u>"No", go to E.5"</u>

F.3 – Where do you keep your "pharmacy remedies" (ask to show the place)?
____
____
____

⇨ IF POSSIBLE, ASK FOR THE MEDICINE CABINET.

F.4 – Characteristics of the place where the "pharmacy remedies" are kept **(interviewer observation)**?
a. Direct contact with sun or sunny place ☐
b. Direct contact with humidity ☐
c. Direct contact with heat ☐
d. Additional observations:
____
____
____

F.5 – How do you identify your "pharmacy remedies"?
a. Color ☐ b. Size ☐ c. Shape ☐
d. Name ☐ e. Box ☐
f. Do not identify them ☐
g. Other ☐**:**______________

F.6 – Do you use to read the leaflet or do you ask someone else to read them for you?
a. **Y** ☐ b. **N** ☐
F.7 – Do you think that "pharmacy remedies" could be risky for your health?
a. **Y** ☐ b. **N** ☐

F.8 – Do you use to look at the validity date of your "pharmacy remedies"?
a. **Y** ☐ b. **N** ☐

F.9 – Have you ever taken an expired "pharmacy remedy"?
a. **Y** ☐ b. **N** ☐ c. **I do not know** ☐

F.10 – When any "pharmacy remedy" has expired, where do you use to throw it away?
____
____

F.11 – Do you use to keep "pharmacy remedies" in their box? a. **Y** ☐ b. **N** ☐

F.12 – Do you use to remove the tablets or pills from the blister to keep them in another place?
a. **Y** ☐ b. **N** ☐

F.13 – Do you use to take advantage of the empty jars of remedies?
a. **Y** ☐ b. **N** ☐

F.14 – Have you used a home remdy during the last month?
a. **Y** ☐ b. **N** ☐ **<u>"No", go to F.18</u>**

F.15 – Which home remedy did you use?
____
____

F.16 – What did you use that home remedy for?
____
____

F.17 – Who told you how to use it?
____
____

F.18 – Each time you feel ill, you prefer to use:
a. Home remedy ☐
b. Pharmacy remedy ☐
c. Both ☐
d. Im do not use anyhting ☐

F.19 – Why?
____
____

F.20 – During the last month, have you taken any remedy or medicine? **a. Y** ☐ **b. N** ☐

"If no medicine was taken, then *<u>GO TO THE NEXT SECTION"</u>*

⇨ IF THE INTERVIEWEE HAD TAKEN ANY PHARMACY REMEDY, ASK HIM/HER TO SHOW THE MEDICAL RECIPE (IF THER IS ONE) AND THE MEDICINE.

⇨ PLEASE FEEL THE BOX BELOW WITH THE INFORMATIONS GIVEN BY THE INTERVIEWEE.

PHARMACY REMEDY (ALLOPATHIC MEDICINE)					
Ord.	F.21	F.22	F.23	F.24	F.25
	Name of the "pharmacy remedy" (allopathic medicine)	Time in use	Who prescribed?	Where did you get it?	What did you use it for?
a	___<br>___<br>___	Days I__I__I<br>Months I__I__I<br>Years I__I__I	(a) Physician ☐ (b) Dentist ☐ (c) Pharmacist ☐ (d) Nurse ☐ (e) CHA ☐ (f) His/her own ☐ (g) Friend/Neighbor ☐ (h) Family ☐ (i) Other ☐:___	(a) Pharmacy ☐ (b) Health Center ☐ (c) Hospital ☐ (d) IMTC ☐ (e) Policlinic ☐ (f) ACS ☐ (g) Friend/neighbor☐ (h) Family ☐ (i) Other ☐:___	___<br>___<br>___
b	___<br>___<br>___	Days I__I__I<br>Months I__I__I<br>Years I__I__I	(a) Physician ☐ (b) Dentist ☐ (c) Pharmacist ☐ (d) Nurse ☐ (e) CHA ☐ (f) His/her own ☐ (g) Friend/Neighbor ☐ (h) Family ☐ (i) Other ☐:___	(a) Pharmacy ☐ (b) Health Center ☐ (c) Hospital ☐ (d) IMTC ☐ (e) Policlinic ☐ (f) ACS ☐ (g) Friend/neighbor☐ (h) Family ☐ (i) Other ☐:___	___<br>___<br>___
c	___<br>___<br>___	Days I__I__I<br>Months I__I__I<br>Years I__I__I	(a) Physician ☐ (b) Dentist ☐ (c) Pharmacist ☐ (d) Nurse ☐ (e) CHA ☐ (f) His/her own ☐ (g) Friend/Neighbor ☐ (h) Family ☐ (i) Other ☐:___	(a) Pharmacy ☐ (b) Health Center ☐ (c) Hospital ☐ (d) IMTC ☐ (e) Policlinic ☐ (f) ACS ☐ (g) Friend/neighbor☐ (h) Family ☐ (i) Other ☐:___	___<br>___<br>___
d	___<br>___<br>___	Days I__I__I<br>Months I__I__I<br>Years I__I__I	(a) Physician ☐ (b) Dentist ☐ (c) Pharmacist ☐ (d) Nurse ☐ (e) CHA ☐ (f) His/her own ☐ (g) Friend/Neighbor ☐ (h) Family ☐ (i) Other ☐:___	(a) Pharmacy ☐ (b) Health Center ☐ (c) Hospital ☐ (d) IMTC ☐ (e) Policlinic ☐ (f) ACS ☐ (g) Friend/neighbor☐ (h) Family ☐ (i) Other ☐:___	___<br>___<br>___
e	___<br>___<br>___	Days I__I__I<br>Months I__I__I<br>Years I__I__I	(a) Physician ☐ (b) Dentist ☐ (c) Pharmacist ☐ (d) Nurse ☐ (e) CHA ☐ (f) His/her own ☐ (g) Friend/Neighbor ☐ (h) Family ☐ (i) Other ☐:___	(a) Pharmacy ☐ (b) Health Center ☐ (c) Hospital ☐ (d) IMTC ☐ (e) Policlinic ☐ (f) ACS ☐ (g) Friend/neighbor☐ (h) Family ☐ (i) Other ☐:___	___<br>___<br>___
f	___<br>___<br>___	Days I__I__I<br>Months I__I__I<br>Years I__I__I	(a) Physician ☐ (b) Dentist ☐ (c) Pharmacist ☐ (d) Nurse ☐ (e) CHA ☐ (f) His/her own ☐ (g) Friend/Neighbor ☐ (h) Family ☐ (i) Other ☐:___	(a) Pharmacy ☐ (b) Health Center ☐ (c) Hospital ☐ (d) IMTC ☐ (e) Policlinic ☐ (f) ACS ☐ (g) Friend/neighbor☐ (h) Family ☐ (i) Other ☐:___	___<br>___<br>___
g	___<br>___<br>___	Days I__I__I<br>Months I__I__I<br>Years I__I__I	(a) Physician ☐ (b) Dentist ☐ (c) Pharmacist ☐ (d) Nurse ☐ (e) CHA ☐ (f) His/her own ☐ (g) Friend/Neighbor ☐ (h) Family ☐ (i) Other ☐:___	(a) Pharmacy ☐ (b) Health Center ☐ (c) Hospital ☐ (d) IMTC ☐ (e) Policlinic ☐ (f) ACS ☐ (g) Friend/neighbor☐ (h) Family ☐ (i) Other ☐:___	___<br>___<br>___

SECTION H
"NUTRITION DATA"

H.1 – How many meals dod you have daily (take into account breakfast, lunch, snacks and dinner)? ___
H.2 – How many times per week do you eat the following foods?

Ord.	FOODS	Nerver or almost never	1x/ month	2 x/ month	1x/ week	2-3 x/ week	4-5x/ week	Daily
**1**	Red meat (beef)							
**2**	Red meat (pork)							
**3**	Red meat (games)							
**4**	Poultry (chicken, hen, duck)							
**5**	Fish							
**6**	Eggs							
**7**	Beans							
**8**	Rice or pasta							
**9**	Bread or flour (mandioca, beiju, rosca)							
**10**	Milk							
**11**	Leaves (lettuce, cabbage, etc)							
**12**	Legumes (carrot, jerimum, beetroot, etc)							
**13**	Tubercles (macaxeira, cará, potato)							
**14**	Fruits or natural fruit juice							
**15**	Açaí							
**16**	Pupunha							
**17**	Tucumã							
**18**	Guaraná natural							
**19**	Castanha							
**20**	Sugar							
**21**	Sweets (chocolates, chewing-gum, lollipop)							
**22**	Processed products (canned meat, sausages, apetizers, etc)							
